# Dual Sensitivity—Potentiometric and Fluorimetric—Ion-Selective Membranes

**DOI:** 10.1021/acs.analchem.1c03193

**Published:** 2021-10-26

**Authors:** Emilia Stelmach, Krzysztof Maksymiuk, Agata Michalska

**Affiliations:** Faculty of Chemistry, University of Warsaw, Pasteura 1, 02-093 Warsaw, Poland

## Abstract

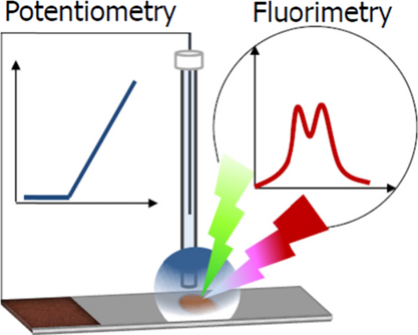

Classical application of ion-selective membranes is limited to either electrochemical or optical experiments. Herein, the proposed ion-selective membrane system can be used in both modes; each of them offering competitive analytical parameters: high selectivity and linear dependence of the signal on logarithm of analyte concentration, high potential stability in potentiometric mode, or applicability for alkaline solutions in optical mode. Incorporation of analyte ions into the membrane results in potentiometric signals, as in a classical system. However, due to the presence of lipophilic positively charged ions, polymer backbones, full saturation of the membrane is prevented even for long contact time with solution. The presence of both positively charged and neutral forms of conducting polymers in the membrane results in high stability of potential readings in time. Optical signal generation is based on polythiophene particulates dispersed within the ion-selective membrane as the optical transducer. An increase of emission is observed with an increase of analyte contents in the sample.

## Introduction

Ionophore-based electrochemical and optical sensors allow precise monitoring of content changes of clinically or environmentally important analytes.^1−3^ The pronounced emphasis of the field of ion-selective sensors is now on high stability and versatility, allowing application for different analytical scenarios, regardless of operation mode. The sensors benefit from ion-selective ionophores embedded with additives in the lipophilic phase; however, there are more differences than common points between optical and electrochemical devices in respect to practice of their application.

Although ion-selective electrodes are equilibrium sensors, in reality, change of the analyte contents in solution affects the outermost region of the membrane.^3,4^ The potential recorded is linearly dependent on logarithm of the activity of analyte ions and typically covers 5–6 orders of magnitude of analyte concentration. On the contrary, ion-selective optodes are most often bulk sensors.^3,5−7^ Optodes’ analytical signal, typically, in a sigmoidal manner is dependent on logarithm of analyte concentration and covers 2–3 orders of magnitude. If incorporation of the analyte into the bulk of the optode is hindered, a linear dependence of optical signals on logarithm of analyte concentration covering a significantly broader concentration range can be obtained, too.^8−10^

Electrochemical sensors, regardless of the construction applied,^2,11,12^ are free to operate in different electrolyte solutions with only limitation set by (usually high) selectivity of the ionophore applied. Electrochemical sensors require the presence of a reference electrode and connection to a voltmeter, which for some applications can be a hindrance. On the other hand, reading signal of ion-selective optical sensors is easier even without complex instrumentations or reference electrodes, e.g., using a mobile phone camera.^13^

As most of the ionophores are optically silent, the optode composition requires the presence of an optical transducer, e.g., a pH-sensitive dye.^5,14^ An alternative approach is use of polyoctylthiophene as an optical transducer.^8,15^

Experimental protocols for potentiometric and optical sensor studies are different. In potentiometric mode, it is generally required that membranes are pre-equilibrated with analyte ions, with the aqueous phase before use. On the other hand, optical sensors, regardless of the applied format, are generally not pretreated before use.^16−18^

The differences in membrane composition/function and pretreatment of potentiometric and optical ion-selective membranes limit applicability of the typical ionophore containing receptors to just one methodology (either optical or electrochemical), which is an obstacle from application point of view. To the best of our knowledge, dual sensitivity ion-selective membranes (DS-ISM) were not reported before. A DS-ISM opens a possibility to use optical readout of electrochemical sensing, allows better understanding of the ion-selective systems, and also improves practical application (e.g., routine testing using different modes than a regular operation one). Last but not the least, DS-ISM—even if used in just one methodology—due to the modified composition can offer, apart from versatility, unpreceded performance.

The natural choice of optical transducer to obtain DS-ISM is poly(octylthiophene). This choice is supported by its well-proven applicability in voltammetric ion sensors,^19^ including potentiometric and optical sensors,^8,9,20−22^ and compatibility with ionophores and ion exchangers.^23−25^

Herein, for the first time, we propose DS-ISM useful both in potentiometric and optical modes, additionally benefiting from improved performance. As a model analyte, potassium ions were chosen.

## Experimental Section

### Reagents

Tetrahydrofuran (THF), poly(vinyl chloride) (PVC), bis(2-ethylhexyl) sebacate (DOS), sodium tetrakis[3,5-bis(trifluoromethyl)phenyl]borate (NaTFPB), valinomycin, regioregular poly(3-octylthiophene-2,5-diyl) (POT), tris(hydroxymethyl)-aminomethane (Tris), potassium hexacyanoferrate(II), and potassium hexacyanoferrate(III) were from Aldrich (Germany).

Other chemicals used, including hydrochloric acid, were of analytical grade and were obtained from POCh (Gliwice, Poland). Doubly distilled and freshly deionized water (resistivity 18.2 MΩ cm, Milli-Qplus, Millipore, Austria) was used throughout this work.

Unless otherwise stated, the buffer used was 0.1 M Tris (adjusted with HCl) to pH 7.3; for a control experiment, 0.1 M Tris buffer (adjusted with NaOH) to pH 9.0 was used.

### Apparatus

In the potentiometric experiments, a Lawson Labs. Inc. instrument (3217 Phoenixville Pike, Malvern, PA 19355, USA) was used, and stable potential readings (potential change <0.5 mV min^–1^) recorded were used to construct calibration graphs. The pump systems 700 Dosino and 711 Liquino (Metrohm, Herisau, Switzerland) were used to obtain sequential dilutions of calibration solutions.

In potentiometric experiments, a double junction Ag/AgCl reference electrode with 1 M lithium acetate in the outer sleeve (Möller Glasbläserei, Zürich, Switzerland) was used. The recorded potential values were corrected for the liquid junction potential calculated according to the Henderson approximation.

In electrochemical measurements, a galvanostat–potentiostat CH-Instruments model 660A (Austin, TX, USA) was used.

Fluorimetric experiments were performed using a spectrofluorimeter (Agilent Technologies, Cary Eclipse). After excitation at a wavelength of 550 nm, the emission intensity was recorded within the range 600–800 nm. Unless otherwise stated, the slits used were 5 nm both for excitation and emission, while the detector voltage was maintained at 1000 V.

To obtain SEM images, carbon fiber paper with or without an ion-selective membrane FE-SEM Merlin (Zeiss) apparatus was used.

The fluorescence visualization of the membrane with POT was performed using a Nikon A1R MP confocal optical microscope.

### Dual Sensitivity Potassium-Selective Membranes

The potassium-selective cocktail contained (in wt%) 4.9% of POT, 9.8% of valinomycin, 2.0% of NaTFPB, 22.0% of PVC, and 61.3% of DOS. A total of 46 mg of membrane components was dissolved in 1 mL of THF. Thus, the mole ratio of POT monomer units to valinomycin to ion exchanger was 10.9:3.8:1. The membrane contained 83.3% w/w of polymers and plasticizers, and the ratio of amounts of plasticizer to PVC was close to 3:1 (by weight). The same cocktail was used to prepare membranes intended for optical and electrochemical studies.

### Preparation of the Potentiometric Paper-Based Sensor

A 2.5 cm × 0.8 cm rectangle was cut from carbon fiber paper (carbon fiber paper PTFE treated, AvCarb Material Solutions) and used as a support for receptor layers. For potentiometric sensors, carbon paper—conductive track—was isolated using PTFE adhesive tape as described previously,^21,26^ leaving an opening of diameter 6 mm for the ion-selective membrane to be applied.

Unless otherwise stated, to the opening in the PTFE foil, 40 μL of potassium-selective cocktail was applied in 5 μL portions, and the cocktail was applied directly on carbon. Between the layers, the applied paper was left to dry at room temperature. The thickness of resulting membranes was equal to 80 ± 2 μm (*n* = 3). For the control experiment, a thin membrane was prepared using a single 5 μL portion of the cocktail.

The tested sensors were not preconditioned before experiments; i.e., the as-prepared sensors were used (as in optical experiments).

### Preparation of the Optical Paper-Based Sensor

For fluorimetric experiments, only the ion-selective membrane parts of the potentiometric sensors were used; i.e., 6 mm diameter circles were cut from carbon fiber paper. The rest of the procedure for receptor layer preparation was the same as that for potentiometric sensors. The as-prepared sensors were placed in the wells of a 96-well plate for fluorescence measurements. The analyte ion solution, optionally in the presence of Tris buffer, was added to the wells.

## Results and Discussion

### Dual Sensitivity Ion-Selective Membrane (DS-ISM)—The Role of POT

To prepare a DS-ISM, the POT conducting polymer was added to the PVC phase. As POT is insoluble in the membrane plasticizer, mixing the polymer with the membrane cocktail results ultimately in formation of particulates of the polymer within the PVC membrane phase.^20,21^ Thus, the interface between the POT and PVC phase is extended, resulting in a more facile ion exchange.^27^ POT particulates in the membrane are isolated from the solution ionic redox species influence,^8^ the effect that was an Achilles heel of unmodified POT sensors.^23,24^ Adding POT to the membrane allows control of the amount of polymer present in the phase and eliminates the spontaneous partition of POT (from the transducer layer) to the membrane phase.^20^

Taking into account that POT present in the membrane interacts with ionophore/ion exchanger,^9,28^ the contents of these components need to be adjusted in the composite membrane. The maximal number of POT^+^ cations formed is dependent on the amount of anion exchanger available (in this work ca. 0.04 M). Thus, the amount of POT^+^ formed is clearly much lower from the maximal doping level close to 25% (total POT monomer unit concentration is ca. 0.46 M).

In aerated solution, spontaneous transformation of neutral polymer backbones, POT^0^, to positively charged ones, POT^+^, occurs to some extent within the membrane at the expense of the oxygen/water redox couple reaction.^8,9,25^ The relatively lipophilic POT^+^ cations formed in the membrane are stabilized by the ionophore, and interactions of the ionophore with POT^+^ are preferred over those with mobile cations of the ion exchanger.^9,28^ Ultimately, the presence of POT^+^ in the membrane will result in a decreased primary ion exchange with solution, similar to that observed earlier for other lipophilic cations.^29^ On the other hand, the presence of the redox couple, POT^0^ and POT^+^, in the membrane is expected to result in an increase of the stability of potential readings in time, similar to that reported previously for other systems.^30−36^

Incorporation of the analyte into the DS-ISM results in potentiometric signal formation, similar to that observed for classical systems and heterogeneous ion-selective membranes; e.g.,^30,33^ optical signal formation requires transformation of POT^+^ (immobilized in the membrane phase) to emission active POT^0^, due to influence of cations on redox equilibrium described earlier, Scheme 1 in the Supporting Information.^1,8,9^

This process can be described by the following simplified reaction, eq 1:
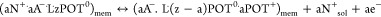
1where L is the ionophore, A^–^ is the cation exchanger, N^+^ is the cation, mem and sol refer to the membrane and solution phase, respectively, and a and z are stoichiometric coefficients; for simplicity, the stoichiometric coefficient of the ionophore is omitted.

Occurrence of reaction (1) requires that PVC domains next to polythiophene particulates contain analyte ions, i.e., dependence on diffusion being the rate-limiting step. Ultimately, for optical signals, a linear dependence of emission, in turn on-mode, on logarithm of analyte concentration in solution is expected.^10^

### Dual Sensitivity Ion-Selective Membrane

Figure S1 shows the SEM image of DS-ISM coated on the carbon paper support and as obtained support for comparison. Application of the membrane cocktail results in a uniform layer formation, covering both carbon fibers and the space between them.

The confocal microscopy image of the surface of DS-ISM post contact with potassium ions, Figure S1, clearly shows that the surface of DS-ISM shows emission from the whole area, proving that POT is well dispersed within the phase.

Figure S2 shows impedance spectra and results of chronopotentiometric studies of DS-ISM sensors. Obtained results are typical for the ion-selective PVC-based membrane, confirming that the conducting polymer is not in direct contact with solution. The DS-ISM is characterized with resistance equal to 7.4 × 10^5^ ohm and capacitance equal to 3.1 × 10^–5^ F, as calculated from the chronopotentiometric experiment.

**Figure 1:**
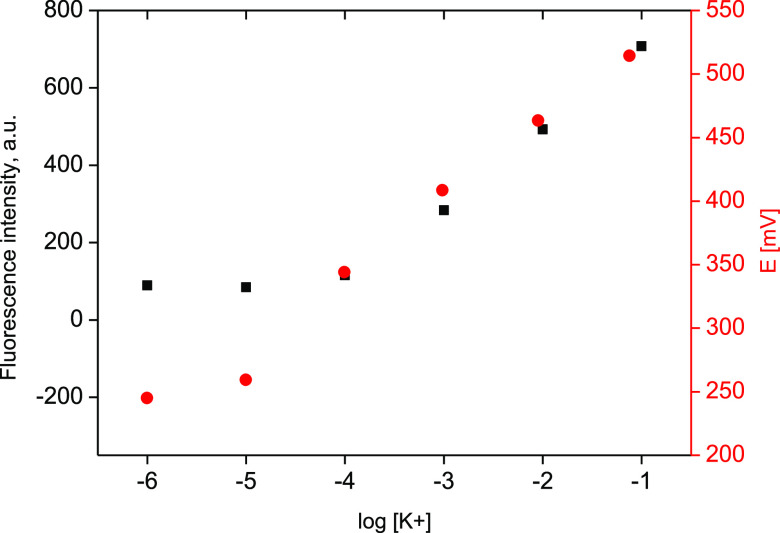
Dependence of the dual sensitivity K^+^-selective membrane on the logarithm of potassium ion concentration change in solution (KCl) recorded in the presence of Tris buffer pH 7.3 (red solid circle) potentiometric mode and (black solid square) fluorimetric mode (emission intensity read at 660 nm).

Under conditions of the fluorimetric experiment, Figure S3, in the absence of potassium ions, emission spectra of polythiophene dispersed in DS-ISM were similar to those of aggregated polymer chains as in POT nanostructures^8,9^ with two maxima, higher at 660 nm and lower at ca. 720 nm, Figure S3. An increase of potassium ion concentration in solution led to an increase of emission at both maxima, i.e., as reported earlier for POT nanoptodes,^8,9^Figure S3. The emission read at the maximum plotted against logarithm of analyte concentration in solution was linear within the concentration range from 10^–4^ M to 0.1 M (*R*^2^ = 0.997), Figure 1. The obtained linear dependence points out to prevalence of diffusion limitation within the ion-selective membrane.^8,10^

In potentiometric mode, for the above given range, the slope of the dependence of potential on logarithm of potassium ion concentration was Nernstian within the range of experimental error and equal to 58.8 ± 1.7 mV/dec (*R*^2^ = 0.998), Figure 1. The concentration change from 10^–4^ to 10^–5^ M resulted in ca. 85 mV shift in potential, as expected for the ISM containing lipophilic interferent cations (positively charged polymer backbones, POT^+^).^29^

**Figure 2:**
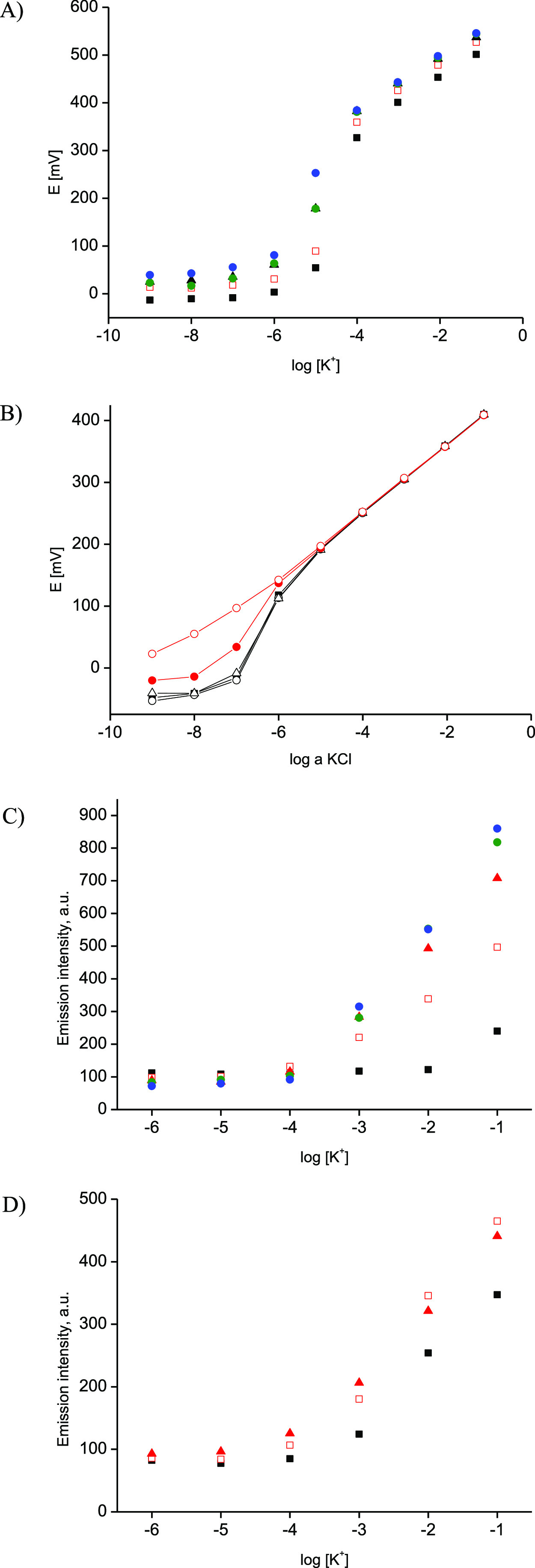
Dependences recorded for K^+^ sensors for different probe–membrane contact times. (A) Potentiometric responses recorded after (black solid square) 0 min, (red open circle) 45 min, (red solid triangle) 90 min, (teal solid circle) 125 min, or (blue solid circle) 160 min of contact with the analyte. (B) Potentiometric responses recorded in KCl after 20 h conditioning of the membrane in 10^–3^ M KCl; three consecutive calibrations are shown (black solid square), (black open circle), and (black open triangle), and then, the sensor was in contact with 1 M KCl for 20 min, and two consecutive calibrations were recorded: (red open circle) first and (red solid circle) the second. C) Emission intensity changes (emission intensity read at 660 nm) as a function of analyte concentration changes recorded after (black solid square) 0 min, (red open circle) 30 min, (red solid triangle) 60 min, (teal solid circle) 90 min, or (blue solid circle) 120 min of membrane contact with the analyte, (D) similar dependence recorded for the thin membrane after (black solid square) 0 min, (red open circle) 30 min, and (red solid triangle) 60 min of contact with the analyte. (Emission and potentiometry experiments have different time scales, and the experimental procedure for optical sensors involved testing one sensor in one solution for a given time, whereas potentiometric dependencies were recorded for one sensor tested in different solutions.)

Figure 2 shows the obtained dependencies recorded in a wider concentration range and their changes in time. For a lower concentration range, different performances are seen under optical and potentiometric conditions. Potentiometric responses, Figure 2A, follow the pattern as expected for the ISM not fully equilibrated with primary ions.^4,16^ Consecutive calibrations are slightly shifted toward higher potential values of ca. 45 mV for a higher concentration range. However, after ca. 90 min contact time of the sensor with analyte-containing solutions, potential readings for the respective concentrations stabilize. For a concentration range from 10^–4^ to 10^–6^ M, a super-Nernstian potential decrease was prevailing.

Assuming that after *t* = 90 min, the membrane is equilibrated with the sample, the thickness of the analyte ion penetration depth in the membrane (*Dt*)^1/2^ can be estimated. Taking into account the diffusion coefficient of monovalent ions in the PVC-based membrane (usually assumed to be close 10^–8^ cm^2^/s^37^), the distance covered is close to 100 μm, corresponding to the membrane thickness. Thus, as expected, occurrence of the super-Nernstian region results from the interactions of positively charged polythiophene backbones with the ionophore, acting as the strongly lipophilic—thus preferred in the membrane phase—interferent.^9,29^

This conclusion is fully supported by the performance of DS-ISM pretreated for 20 h in 10^–3^ M KCl. For the KCl concentration range from 10^–1^ to 10^–5^ M, the slope of dependence was close within the range of experimental error to Nernstian 55.6 ± 0.5 mV/dec (*R*^2^ = 0.999) followed by abrupt potential change for lower concentrations. Contact of the sensor with 1 M KCl for 20 min resulted in transient disappearance of the super-Nernstian region on potential vs logarithm of activity on the very first dependence recorded; however, the abrupt potential decrease was restored on the consecutive calibration curve. This result fully supports claims that the DS-ISM membrane is permanently not saturated with potassium ions. The observed potentiometric responses are related to exchange of analyte ions between the solution and relatively thin surface-most layer of the membrane.

Uniquely for the primary-ion nonsaturated membrane, the potentials recorded in the linear response range (10^–4^–0.1 M) were characterized with excellent stability. The standard deviation (SD) of potential values recorded for KCl concentrations ≥10^–4^ M did not exceed 0.8 mV (*n* = 5, including traces recorded directly after contact with 1 M KCl, where memory effects are expected to be the most severe). Slightly higher changes were observed for 10^–5^ M KCl, with SD equal to 1.2 mV. The high stability of potential readings observed is attributed to the unique feature of the herein presented membrane—the presence of both positively charged and neutral polymer backbones of POT in the membrane.

Dependence of emission signals recorded on logarithm of concentration of KCl in solution is shown in Figure 2C. Similar to the case of electrochemical mode, the recorded dependencies were affected by sample–probe contact time; with sample contact time elapsing, higher intensities were obtained. Starting from 30 min contact time, neither the linear response range nor the detection limit was affected; the linear dependence of emission on logarithm of concentration of KCl was observed within the range from 10^–4^ to 0.1 M, (*R*^2^ = 0.997, for 60 min), Figure 2C. However, if thinner membranes were used, Figure 2D, the increase in observed emission was much faster, as expected for diffusion in the membrane-controlled process.

It should be stressed that fluorimetrically active polythiophene nanostructures respond to change in potassium ions present in the membrane. Because the rate of ion incorporation into the membrane is linearly dependent on potassium ion concentration in the sample, the amount of ions incorporated to the nonsaturated membrane will also be linearly dependent on ion concentration in solution. Therefore, the recorded fluorimetric signal, which is directly proportional to logarithm of ion concentration in the membrane, is also linearly dependent on logarithm of ion concentration in solution. However, for a longer contact time sufficient to saturate the membrane, potassium ion concentration in the membrane approaches its maximum value (for defined conditions) and becomes independent of potassium ion concentration in the solution.

For short contact time of the membrane with the sample, the membrane is not saturated. Therefore, the response in both modes is directly determined by various effects: sample concentration in potentiometric mode and membrane bulk concentration in fluorimetric mode.

Figure S4 shows potential and emission signal dependence obtained for three nominally the same sensors (for the optical approach after 1 h contact time with the analyte). Results shown in Figure S4 clearly confirm that SD both in the potentiometric and optical approach is relatively small taking into account manual sensor preparation (≤7%). Slightly higher absolute SD values were obtained in the optical approach; however, it should be stressed that due to the different nature of the technique, individual traces recorded were characterized with a somewhat higher noise.

Taking into account limitation in analyte incorporation into the DS-ISM, it should be underlined that the permselectivity of the DS-ISM was fully confirmed—as expected, the 20 h long experiment performed in KCl, KNO_3_, and K_2_SO_4_ led to similar linear dependencies (results not shown).

### Resistivity to Interferences

The sensitivity of herein proposed systems for redox potential changes was tested both in electrochemical and optical mode, Figure S5. Figure S5A shows potentiometric dependence obtained for the support used—carbon fiber paper (potential values recorded are given in Table S1). The paper in the absence of the membrane (Figure S5A) shows slightly lower than Nernstian, yet pronounced, dependence of potentials recorded on solution redox potential, with the slope equal to 51.4 ± 0.6 (*R*^2^ = 0.999). However, after coating the herein proposed ion-selective membrane, recorded potential values were independent of the change of solution redox potential (values are given in Table S1).

In optical mode, the emission spectra recorded were not affected by the change of solution redox potential, Figure S5B, similar to that previously observed for nanoptodes based on POT.^8^ The observed insensitivity of DS-ISM to change of the solution redox potential is clearly related to the presence of the PVC-based matrix; however, stabilization of POT^+^ by the ionophore can also contribute to this effect.

Selectivity for cation interferences was tested both in potentiometric and optical mode. As shown in Figure S6A, in potentiometric mode, linear responses of potential vs logarithm of interferent ion activity changes in solutions were observed, as expected for the super-Nernstian region showing sensors. Logarithms of selectivity coefficients obtained for model interferents, Na^+^, H^+^, Mg^2+^, and Ca^2+^ ± SD, were equal to −5.4 ± 0.1, −7.5 ± 0.2, −8.1 ± 0.3, and −7.3 ± 0.3, respectively. As shown in Table S2, obtained log *K*_sel_ values are significantly lower, more favorable, compared to those typically characterizing potentiometric sensors, regardless of the construction applied.^38,39^ This effect is attributed to the presence of POT^+^ ions in the membrane structure and limited exchange of other ions between the membrane and solution.

Optical selectivity is presented in Figure S6B; despite the emission increase observed for increasing KCl solution, in sodium, hydrogen, or magnesium ion solution, an emission increase, beyond the range of experimental error, was not observed for the increasing interferent ion concentration. For calcium ions, an increase of concentration to 10^–2^ and 0.1 M resulted in some increase in emission, yet much lower compared to equivalent change in potassium ion concentration. Thus, the proposed DS-ISM offers high selectivity in both modes.

**Figure 3:**
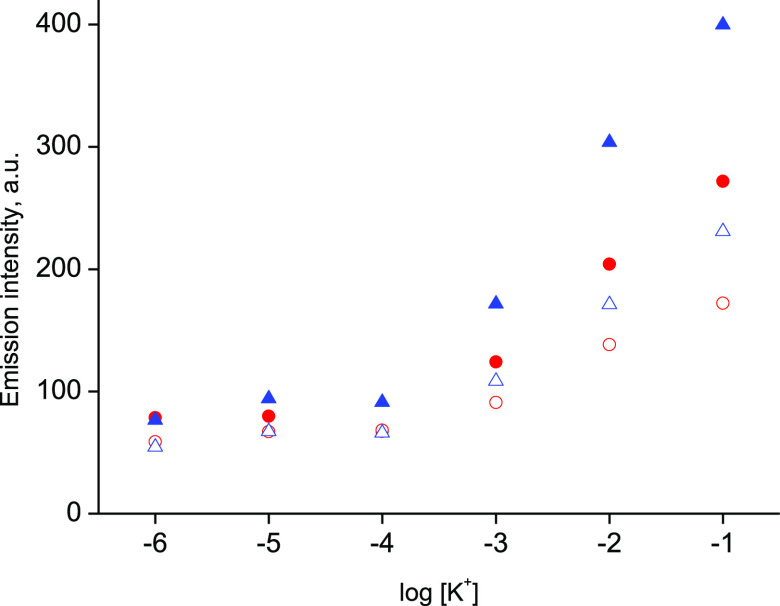
Effect of sample pH on the performance of optical sensor: emission intensity changes (emission intensity read at 660 nm) after (red solid circle/red open circle) 30 min and (blue solid triangle/blue open triangle) 60 min of contact with the analyte at (red solid circle/blue solid triangle) pH 9.0 and (red open circle/blue open triangle) pH 7.3; for this experiment, voltage at the fluorimeter detector was set lower compared to results shown in Figure 2, to allow observation of responses both at pH 9.0 and 7.3 under the same experimental conditions.

Classical optode systems applying pH-sensitive dyes as optical transducers are typically limited with applicability to the pH range close to 7;^1,14^ however, the herein proposed system as shown in Figure 3 also yielded emission change for analyte concentration change at significantly higher pH. It should be stressed that at pH equal to 9.0, higher emission intensities were observed compared to results obtained at pH = 7.3 (for the same experimental conditions) (Figure 3). Thus, the results shown in Figure 3 clearly support unique benefits of DS-ISM application in optical mode.

### Reversibility

Figure S7A shows potentiometric trace of increasing and decreasing concentrations of KCl of the DS-ISM. The dependencies recorded in the experiment time scale corresponding to the electrochemical test, Figure S7A, clearly show that the potentiometric sensor is fully reversible.

Optical responses recorded in the time scale typical for this experiment (after 30 min contact time of the sensor with solution) show that emission intensities observed in response to contact of the sensor with the analyte are still observed, even if the sample is transferred to buffer solution free of analyte. However, it should be stressed that another contact with a “fresh” portion of primary ions results in a further increase of emission—the sensors operate in “cumulative” mode. This result also shows that the membrane is not saturated with primary ions during contact with the initial sample, supporting previous conclusions. The observed effect can be attributed to high sensitivity of POT in emission changes to change of oxidized and reduced polymer backbone ratios^40^ and high affinity of ionophore–K^+^ or ionophore–POT^+^ interactions in the PVC membrane phase.

## Conclusions

In this work, a dual sensitivity ion-selective sensor was proposed. The same composition of ion-selective membranes shows a linear dependence of analytical signals: emission increase or potential increase with an increase of analyte concentration, under fluorimetric or potentiometric conditions, respectively. In either mode, the dual sensitivity membrane-based sensor offers a linear dependence of signal vs logarithm of potassium concentration in samples within the range from 10^–1^ to 10^–4^ M. The proposed sensors benefit from the presence of oxidized and neutral polyoctylthiophene backbones dispersed within the PVC-based membrane phase. The presence of POT contributes to unique properties of the proposed system—optical sensitivity, exceptionally high selectivity, and unique stability of potential readings in time. The behavior in the potentiometric and fluorimetric response range results generally from different response mechanisms ruling sensitivity to primary ion activity in the solution (potentiometric mode) or in the membrane (optical mode). The herein proposed system offers unique possibility of comparison of mechanisms of ion-selective membrane operation and is potentially attractive for applications.

## Supporting Information Available

Scheme of the mechanism of electrochemical and optical signal generation, confocal image of obtained sensors, impedance spectra and results of chronopotentiometric studies obtained, emission spectra of POT dispersed in the membrane, potential and emission signal dependence obtained for three nominally the same sensors, optical and electrochemical responses to redox interferents present in solution, potential values recorded for the support in the presence of the redox couple in solution, selectivity, obtained logarithms of selectivity coefficients, and reversibility of recorded responses (PDF)
