# Supplementary material for: Dual Sensitivity—Potentiometric and Fluorimetric—Ion-Selective Membranes
Source: Anal Chem. 2021 Oct 26;93(44):14737–42. doi: 10.1021/acs.analchem.1c03193 (PMC8581967; doi:10.1021/acs.analchem.1c03193)
Supplement: Supplementary file 1 — ac1c03193_si_001.pdf [file ac1c03193_si_001.pdf]

Supporting information

Dual sensitivity - potentiometric and fluorimetric - ion-selective membranes

Emilia Stelmach, Krzysztof Maksymiuk, Agata Michalska*

Faculty of Chemistry, University of Warsaw, Pasteura 1, 02-093 Warsaw, Poland

* Corresponding author: agatam@chem.uw.edu.pl, tel. +48 22 55 26 331

Table of Content

1. Scheme of mechanism of electrochemical and optical signal generation.

2. Images of carbon paper used and the material covered with ion-selective membrane – SEM and confocal microscopy.

3. The results of electrochemical impedance spectroscopy and chronopotentiometric experiments performed for the proposed sensor.

4. Dependence of emission spectra of optical sensor on changes of KCl solutions concentration.

5. Mean value ± SD calculated from dependencies recorded for 4 nominally the same sensors in KCl solutions within concentration range from 10^{-1} to 10^{-4} M in potentiometric mode (without pretreatment) and in optical mode.

6. Effect of solution redox potential change on electrochemical and optical signal of dual sensitivity ion-selective membranes.

7. Selectivity of dual sensitivity ion-selective membranes tested in model interferents in potentiometric and in optical mode.

8. Reversibility of response of potassium-selective dual sensitivity sensor in potentiometric and in optical mode.

9. Values of potential recorded for tested sensor in redox buffers of different redox potential.

10. The logarithms of selectivity coefficients (log $K_{K,\ J}$) ± SD obtained for proposed sensors.

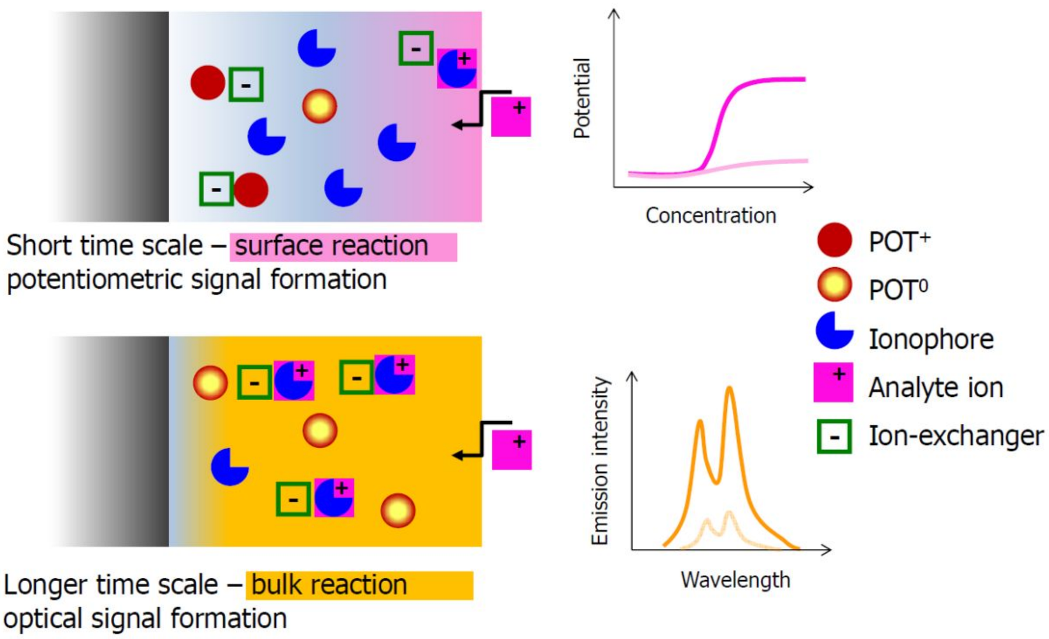

Scheme 1. Scheme of mechanism of electrochemical and optical signal generation.

A)

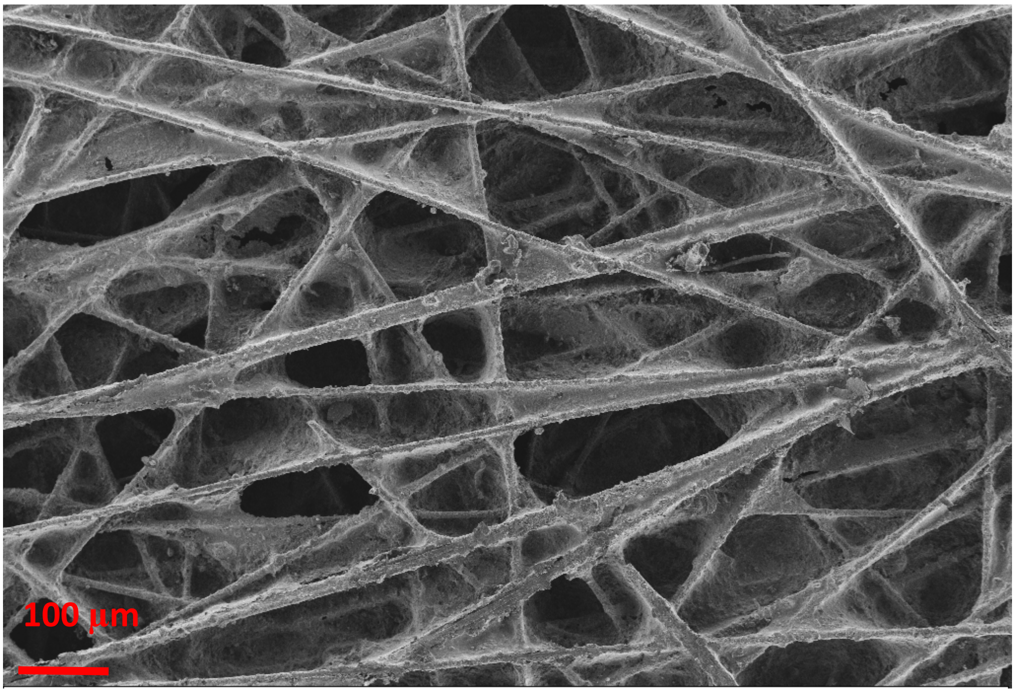

B)

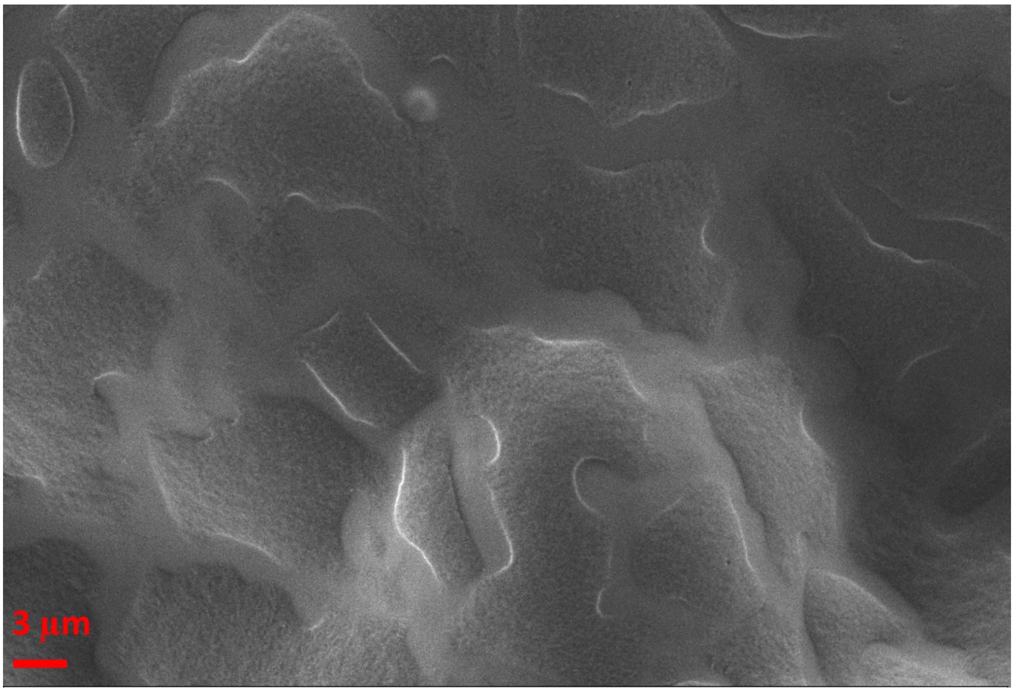

C)

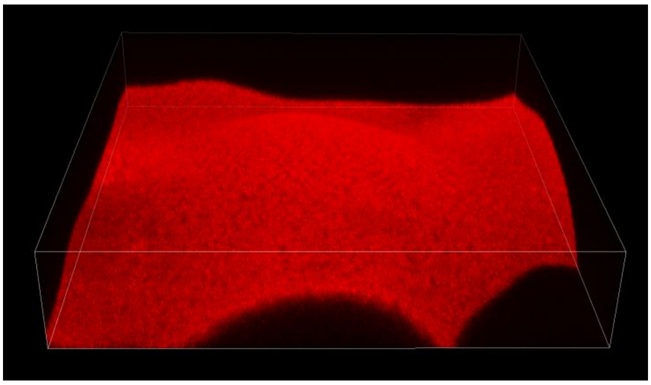

Figure S1. A) and B) SEM images of A) as obtained Carbon Fiber Paper (without ion-selective membrane) and B) Carbon Fiber Paper covered with dual sensitivity ion-selective membrane. C) Confocal image of surface dual sensitivity ion-selective membrane post contact with potassium ions.

A)

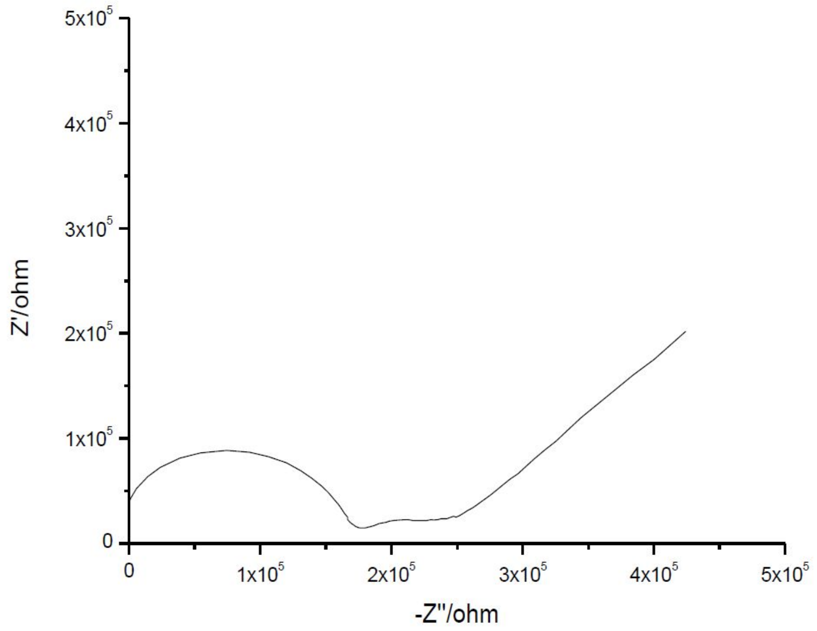

B)

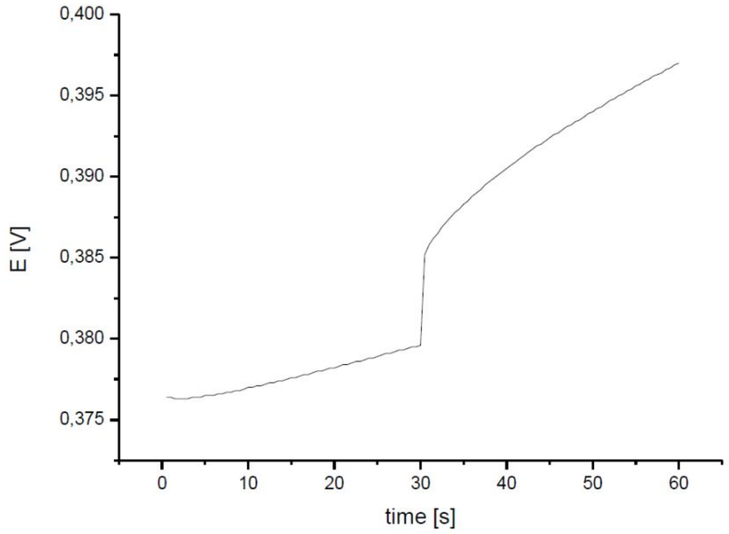

Figure S2. A) The results of electrochemical impedance spectroscopy experiments performed for proposed sensor in 0.1 M KCl within the frequencies range from 0.01 to 10^5 Hz at E = 0.3 V with amplitude 50 mV. B) The results of chronopotentiometric experiments applying cathodic/anodic current of 10 nA, in 0.1 M KCl.

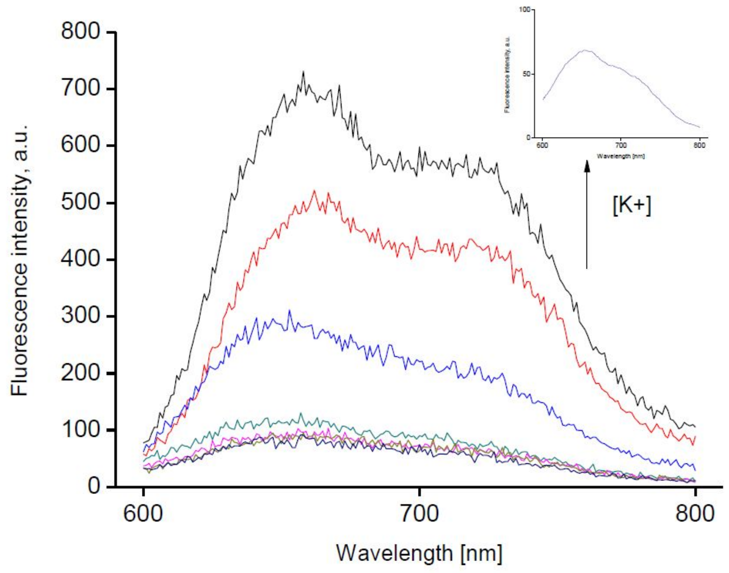

Figure S3. Dependence of emission spectra of optical sensor on changes of KCl solutions concentration within the range from 0.1 to 10^{-6} M in the presence of Tris buffer pH= 7.3, dependences were recorded for contact time with samples equal to 1 h (excitation wavelength = 550 nm, slits 10/10 nm, detection voltage = 1000 V). Inset: magnification of emission spectra of DS-ISM recorded in the absence of potassium ions in sample.

A)

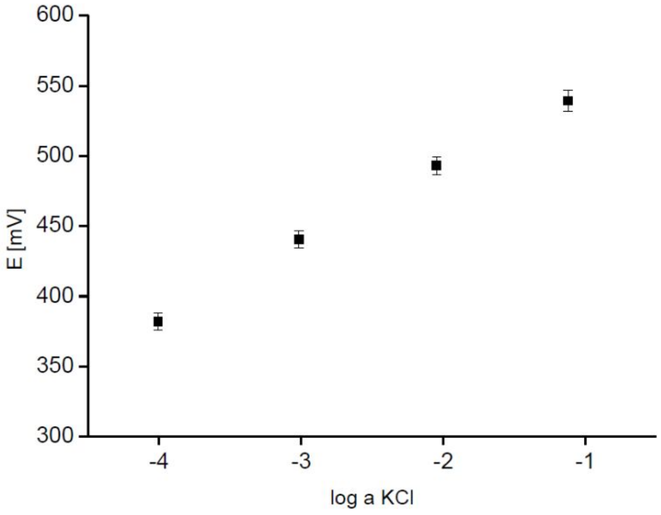

B)

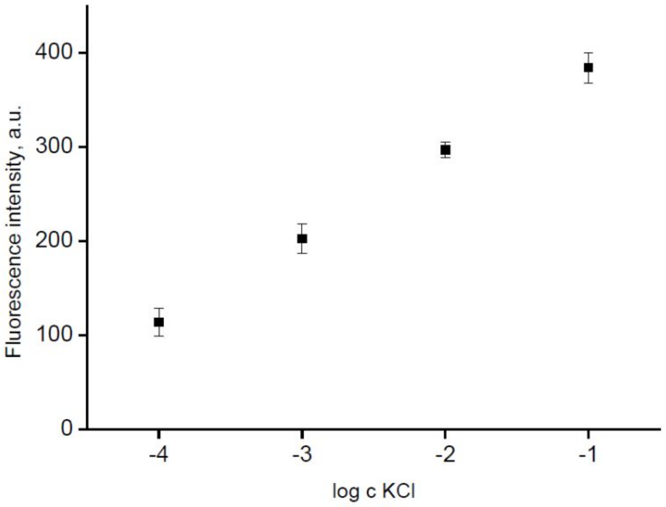

Figure S4. Mean value ± SD calculated from dependencies recorded for 4 nominally the same sensors in KCl solutions within concentration range from 10^{-1} to 10^{-4} M: A) in potentiometric mode (without pretreatment) and B) in optical mode.

A)

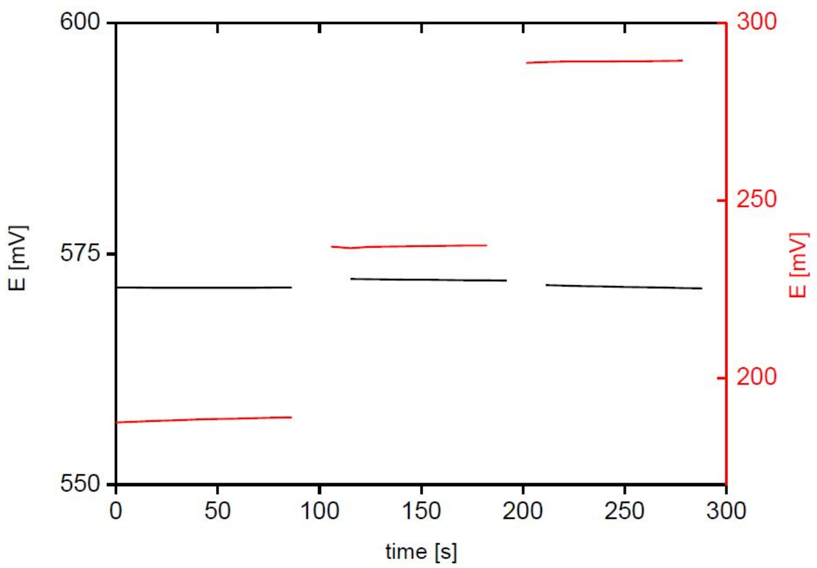

B)

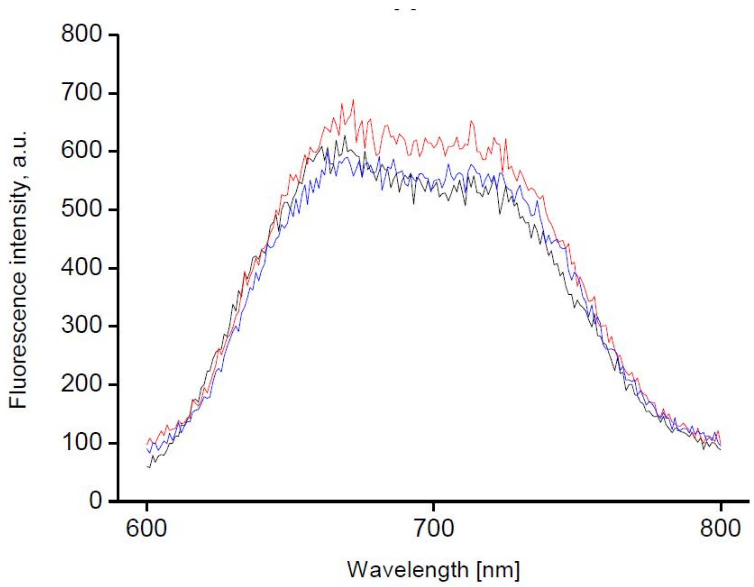

Figure S5. Effect of solution redox potential change on A) electrochemical and B) optical signal of dual sensitivity ion-selective membranes. A) Potentiometric response recorded for (black line) dual sensitivity sensor and for comparison (red line) for Carbon Fiber Paper support used (in the absence of ion-selective membrane) recorded in the presence of redox buffers of different redox potential (potassium hexacyanoferrate(III) and potassium hexacyanoferrate(II)) of equal concentrations 0.1 M mixed in the volume ratio 1:10, 1:1 and 10:1 in the presence of 0.1 M KCl, note the y-axis scale difference for black

and red traces, shown. B) Emission spectra recorded for dual mode sensor in the presence of redox buffers of different redox potential (potassium hexacyanoferrate(III) and potassium hexacyanoferrate(II)) of equal concentrations 0.1 M mixed in the volume ratio (blue line) 1:10, (red line) 1:1 and (black line) 10:1 in the presence of 0.1 M KCl.

A)

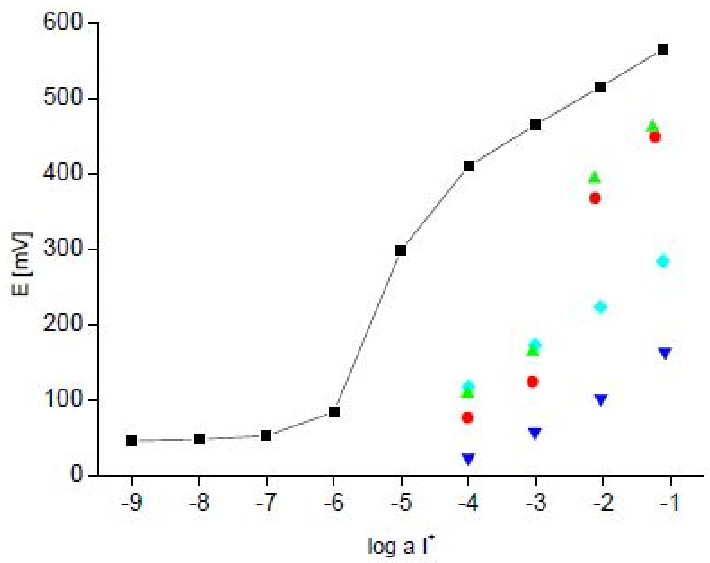

B)

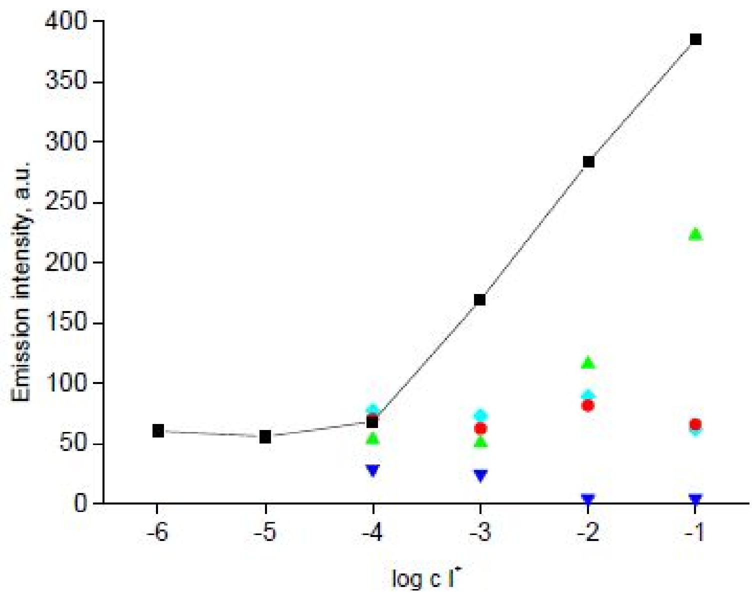

Figure S6. Selectivity of dual mode ion-selective membranes tested in model interferents, for comparison responses recorded in potassium ions solutions are included: A) potentiometric mode and B) optical – emission mode, sensors performance was tested in: (■) KCl, (▲) $CaCl_2$, (●) $MgCl_2$, (♦) NaCl and (▼) HCl solutions.

A)

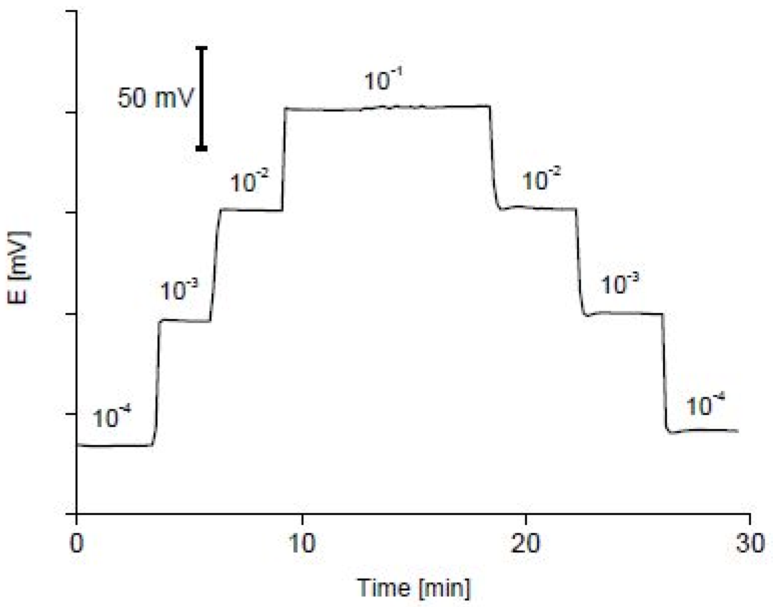

B)

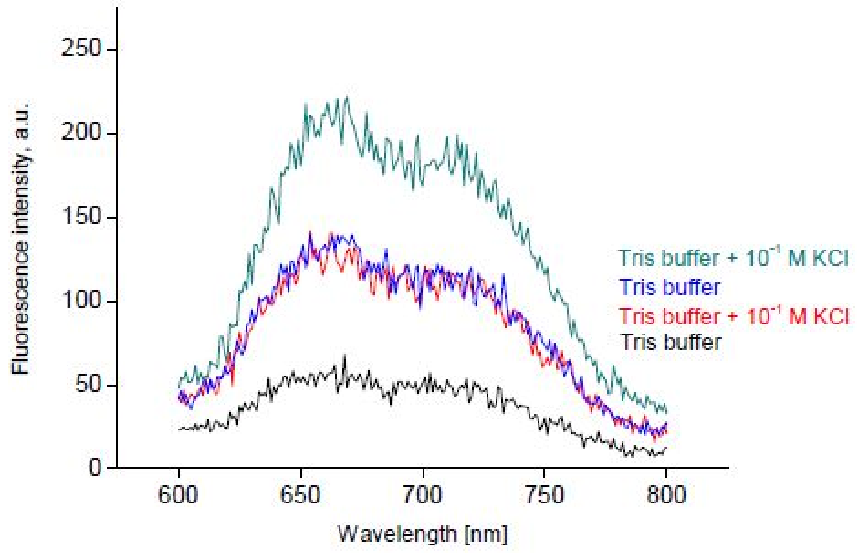

Figure S7. Reversibility of response of potassium-selective dual sensitivity sensor in A) potentiometric – KCl solutions of increasing and then decreasing concentrations within the range from 10^{-4} to 10^{-1} M, B) optical mode – emission spectra recorded in Tris buffer or Tris buffer in the presence of 0.1 M KCl, each curve recorded after 30 minutes sensor – solution contact time.

Table S1. Values of potential recorded in redox buffers of different redox potential (potassium hexacyanoferrate(III) and potassium hexacyanoferrate(II)) of equal concentrations 0.1 M mixed in the volume ratio 1:10, 1:1 and 10:1 recorded for dual sensitivity membrane potassium selective sensor.

Log [Red/Ox]	E [mV]
1	571,5
0	572,1
-1	571,6

Table S2. The logarithms of selectivity coefficients (log $K_{K, J}$), mean ± SD, for activities range from 0.1 to 10^{-4} M or 10^{-3} to 10^{-4} M in case of Mg^{2+} and Ca^{2+} ions (separate solution method) obtained for proposed sensors, the values were determined using slopes of experimental electrodes characteristics), for comparison literature data are included [1].

Interefering ion	Log $K_{K, J}$ ± SD	Ref.[1]
Na^{+}	-5.4 ± 0.1	-3.1 ± 0.1
Mg^{2+}	-8.1± 0.3	-3.4 ± 0.4
Ca^{2+}	-7.3± 0.3	-3.4 ± 0.4
H^{+}	-7.5 ± 0.2	-4.5 ± 0.3

References

[1] Jaworska, E.; Lewandowski, W.; Mieczkowski, J.; Maksymiuk, K.; Michalska, A. Critical assessment of graphene as ion-to-electron transducer for all-solid-state potentiometric sensors. *Talanta* **2012**, *97*, 414–419.
